# Impact of COVID-19 Pandemic and Lockdown Measures on the Mental Health of the General Population in the Gulf Cooperation Council States: A Cross-Sectional Study

**DOI:** 10.3389/fpsyt.2021.801002

**Published:** 2021-12-20

**Authors:** Naif Al-Mutawa, Nourah Al-Mutairi

**Affiliations:** ^1^Department of Community Medicine, Faculty of Medicine, Health Sciences Center, Kuwait University, Kuwait City, Kuwait; ^2^Department of Medicine, Faculty of Medicine, Health Sciences Center, Kuwait University, Kuwait City, Kuwait

**Keywords:** COVID-19, depression, anxiety, GCC countries, post-traumatic stress, insomnia

## Abstract

**Background:** In the Gulf Cooperation Council (GCC) countries (Kuwait, Qatar, Saudi Arabia, Bahrain, United Arab Emirates, and Oman), as in the rest of the world, the COVID-19 has been spreading since 2019, and it had a significant impact on various aspects of life. The outbreak and the restrictive measures imposed by countries to stop the spread of the virus could harm the mental health condition of the general population. This cross-sectional study aims to assess the impact of the pandemic on mental health and investigate the potential risk factors.

**Methods:** An online survey was collected from individuals in GCC countries from May to October 2020. The final sample included 14,171 participants, 67.3% females and 60.4% younger than 35 years old. The survey consisted of depression, Anxiety, Insomnia, and post-traumatic stress questionnaires. Crude and adjusted Odds ratios are calculated using simple and multivariable logistic regressions to investigate the association between risk factors and mental health issues.

**Results:** Endorsement rates for depression were 11,352 (80.1%), 9,544 (67.3%) for anxiety, 8,845 (63.9%) for insomnia and 9,046 (65.2%) for post-traumatic stress. Being female and younger age were associated with a higher likelihood of developing depression, anxiety, insomnia, and post-traumatic stress. In addition, participants with underlying psychological problems were three times more likely to develop depressive and post-traumatic stress symptoms.

**Conclusion:** According to the findings, women, youth, singles, divorced individuals, and individuals with pre-existing psychological and medical conditions are subject to a higher risk of mental health problems during the pandemic, which policy-makers should consider when imposing restrictive measures.

## Introduction

The outbreak of Coronavirus at the end of the year 2019 has affected various aspects of life around the world. This Coronavirus (SARS-CoV-2), which causes the highly infectious disease called COVID-19, has spread from Wuhan, China (1) to Southeast Asia, Europe, and the rest of the world (2) and has many similarities to other Coronaviruses like Severe Acute Respiratory Syndrome (SARS) and Middle East Respiratory Syndrome (MERS). It is mainly transmitted via airborne droplets. The disease can be accompanied by fever, cough, shortness of breath, loss of taste, diarrhea, and fatigue. In some cases, it can lead to a severe form of pneumonia, and subsequently, death (3). This pandemic created a considerable workload for medical care professionals worldwide, which led to extreme fatigue, mental distress, and sleeping problems (4). During the COVID-19 Pandemic, several countries implemented restricting measures to prevent the disease from spreading. Several countries imposed strict lockdown regulations, while countries like the USA (5) and Brazil (6) recommended their citizens stay home.

Like other parts of the world, GCC (Gulf Cooperation Council) Arab Countries were also affected by this pandemic. The first confirmed case in GCC countries was announced in UAE on February 2 of 2020. In total, as of August 28, 2021, there have been 2,472,827 confirmed cases and 19,001 deaths in GCC countries (7). As soon as cases of COVID-19 were confirmed, they initiated restrictive measures to slow the spread of infection.

In May 2020, while the governments worldwide were focusing on the infectious aspect of the pandemic, the Director-General of the World Health Organization (WHO), Tedros Adhanom Ghebreyesus, stated that the impact of the pandemic on people's mental health was already extremely worrying (8). This observation has also been supported scientifically since the beginning of the pandemic (9). A steady increase in the evidence on mental health issues during the COVID-19 pandemic from Chinese and Southeast Asian cross-sectional studies showed that not only were the health care workers at risk (10, 11), but also anxiety, depression, and insomnia symptoms were also increased among the public (12–14). The disease itself, the governmental measures like lockdowns, and the lack of effective treatment have led to emotional, psychological, and behavioral changes (15, 16). These changes can manifest themselves as anxiety, depression, and decreased sleep quality. Santini et al. (17) showed that such measures could lead to social isolation and loneliness.

In May 2020, as the number of COVID-19 cases increased in GCC countries, the authorities decided to increase the restrictive measures. However, depending on the country, the scope of these measures was very different. While Bahrain and Qatar opted for a relatively milder approach, with eased flight restrictions and no curfews, other Gulf states imposed more stringent measures with curfews and lockdown in major cities. When this study began, the first epidemic wave in GCC countries had nearly reached its peak. According to Wang et al. (13), it is very likely that the psychological impacts of the lockdown and the disease itself had already emerged by that time.

This study investigates the prevalence of anxiety, depression, insomnia, and post-traumatic distress and their severity level among the general population of GCC countries. In addition, the association between the sociodemographic characteristics and mental health issues during the pandemic and lockdowns is investigated.

## Research Methodology

### Study Design

This is a cross-sectional study, where a web-based survey design is adopted. Approval for this study was obtained from the Health Sciences Centre (HSC) ethical committee of Kuwait University. In addition, electronic informed consent was obtained from each participant, and participants could withdraw from the survey at any time without providing any reason. The survey was conducted from May 21 to November 4, 2020.

#### Sample Size

Based on United Nations' report on Worldometer Website (18), the population of GCC countries was approximately 58,664,098 as of May 2020. Considering the population size of 58,664,098 with a response distribution of 50%, 95% confidence level, and 1.1% margin of error, the minimum required sample size was estimated to be 7,936. The required sample size was calculated using Raosoft® Online Software (19).

#### Data Collection

The survey was administered via Survey Monkey Website (www.surveymonkey.com), and the questionnaires were sent out through social media (including Twitter and Instagram), using convenience and snowball sampling methods. In addition, Twitter and Instagram Ads services were used to reach random participants who were living in one of the GCC countries. In order to maximize the inclusiveness, no exclusion criteria are imposed on sociodemographic factors. However, only participants who were living in one of the GCC countries were included in the final analysis. Twitter and Instagram Ads services were also set up in such a way that only people in these territories were targeted.

### Questionnaires

The survey consisted of two parts. First, the information about sociodemographic characteristics, e.g., gender, age, occupation, marital status and education, physical activity, smoking habit, COVID-19 infection, and the existence of any underlying disease, were collected. Then, the psychological impacts of COVID-19 and lockdowns were obtained using the following questionnaires:

#### Generalized Anxiety Disorder-7

The Arabic version of GAD-7 (Generalized Anxiety Disorder-7), seven items, 4-point Likert scale ranging from 0 = not at all to 3 = almost every day, was used to determine the participant's self-reported magnitude of anxiety symptoms (20). The questionnaire consists of questions about anxiety, irritability, being worried about different things, feeling restless, constant fatigue, muscle rigidity, and sleep and concentration difficulties (21, 22). The total scores of ≥5, ≥10, and ≥15 were rated as mild, moderate, and severe generalized anxiety symptoms. The GAD-7 has had acceptable psychometric validity and reliability (23). Additionally, as measured by Cronbach's alpha (0.80), a recent study on University students in Saudi Arabia found an adequate internal consistency in the Arabic version of GAD-7 (24).

#### Patient Health Questionnaire-9

The PHQ-9 questionnaire consists of nine questions regarding depression. It was developed as a self-administered screening instrument for routine depression diagnosis in somatic medicine (25). Each question score ranges from 0 = not at all to 3 = almost every day (26). AlHadi et al. (21) demonstrated that the Arabic version of the PHQ is a valid and reliable tool to diagnose depression.

#### Insomnia Severity Index

To diagnose insomnia and its severity, the Arabic version of Insomnia Severity Index (ISI) was used, which was translated by Suleiman et al. (27). Suleiman et al. (27) demonstrated that the Arabic version of ISI-7 has adequate reliability and validity for insomnia diagnosis. The ISI-7 is a self-report scale composed of SEVEN items. Each item is evaluated on a 4-point Likert scale (from 0 = not at all to 4 = extremely). The total score ranges from 0 to 28. Twenty eight represents the highest insomnia severity, while 0 indicates that the patient has no insomnia. According to Morin et al. (28), a total score of 0–7 indicated no insomnia, 8–14 indicated mild form of insomnia, 15–21 indicated moderate insomnia, and 22–28 indicated severe insomnia. In this survey, a total score of ISI ≥ 8 indicated insomnia (29).

#### Impact of Event Scale-Revised

The Impact of Event Scale-revised form (IES-R) (30) is a self-administered, 22-item questionnaire. It records typical forms of individual reactions or symptoms to highly stressful events. The consequences of extreme events, e.g., sexual violence acts, war experiences, natural disasters, or one's life-threatening illnesses, have shown three typical psychological reactions recorded by the IES-R: Intrusions (eight-items), Avoidance (eight-items), and Hyperarousal (six-items). Intrusions are intrusive, powerful memories beyond an individual's control that overwhelm working memory with trauma-related images or other sensory impressions. Post-traumatic avoidance behaviors manifest when concerned about giving a wide berth to thoughts, activities, or related situations to trauma and evoking memories. The Hyperarousal symptoms include sleep or concentration disorders, increased irritability, anxiety, and exaggerated startle reactions. The items are rated on a 5-point scale ranging from 0 (“not at all”) to 4 (“extremely”). The IES-R yields a total score ranging from 0 to 88 (31). In this study, the Arabic version of IES-R-22 is used, which was developed and underwent a validity and reliability check by Davey et al. (32).

### Statistical Analysis

Descriptive statistics were used to report the sociodemographic, health, and pandemic-related characteristics of the sample population. In addition, the prevalence of severity of each mental health issue is calculated. Subsequently, the depression, anxiety, insomnia, and post-traumatic stress scores were recategorized as a dichotomous variable. Those with mild to severe symptoms were categorized as “1: Having symptoms” and those without any sign of symptom as “0: Having no symptoms.” Bivariate logistic regressions were used to calculate the Crude Odds Ratios (COR) for all predictor variables, including country, age, gender, marital status, health insurance, level of education, occupation, physical activity, smoking, underlying medical and psychological conditions, duration of lockdown and COVID-19 infection. However, the problem with the bivariate analysis is that it does not account for possible confounding effects. Thus, multivariable logistic regressions were done using the binary logistic regression option in SPSS so that the effect of each explanatory variable can be investigated while controlling for other independent variables. For each mental health condition (depression, anxiety, insomnia, and post-traumatic stress), a separate multivariable logistic regression is conducted, and the adjusted Odds Ratios (AOR) were reported. Finally, a 95% Confidence Interval (CI) was applied to estimate the precision of calculated ORs. The significance level of the statistical tests was 0.05. All statistical analyses were conducted using IBM SPSS 26 (IBM Corp., Armonk, NY, USA) for Windows and Excel MS 2019 (Microsoft Corp., Redmond, Washington, USA).

## Results

### Descriptive Statistics

#### Sociodemographic, Health and Pandemic-Related Characteristics

Sociodemographic information about the sample population is shown in Table 1. Fourteen thousand and seven hundred and eight individuals who participated in this survey. Two hundred and five participants did not complete the survey, and 332 gave inconsistent answers to the questions. In total, the answers of 14,171 participants were included in this study. A large proportion (64.2%; 9,099) of participants were from Kuwait. The others were from five other participating countries. Of the 14,171 individuals, 9,536 (67.3%) were females, and 4,635 (32.7%) were males. 48.5% were married, 44.9% were single, and 5% were divorced. Most respondents who participated in this survey were between 25 and 34 years old. In summary, 3.9% of participants were younger than 18; 24.9% between 18 and 24, 31.6% between 25 and 34, 22% between 35 and 44, and 12.1% between 45 and 54 years old. 5.5% of participants were older than 54. About half of the participants had a Bachelor's degree (49.5%), 18.8% had a high school diploma, and 14.9% had an associate degree. Around 11.7% had a Master's degree, and 5% did not finish high school.

**Table 1:** Descriptive statistics of sociodemographic characteristics including country of residence, gender, age, marital status and level of education, occupation, physical activity, smoking habit, and other health and pandemic related factors.

**Sociodemographic Variables**	**Frequency (N)**	**Percentage (%)**
Country		
UAE	946	6.7
Kuwait	9,099	64.2
KSA	909	6.4
Oman	1,383	9.8
Qatar	811	5.7
Bahrain	1,023	7.2
**Total**	**14,171**	
Gender		
Female	9,536	67.3
Male	4,635	32.7
Age		
<18	554	3.9
18–24	3,535	24.9
25–34	4,472	31.6
35–44	3,123	22.0
45–54	1,708	12.1
55–64	671	4.7
> 65	108	0.8
Marital status		
Married	6,876	48.5
Single	6,359	44.9
Widowed	115	0.8
Divorced	705	5.0
Separated	116	0.8
Level of education		
Less than High School	713	5.0
High School Diploma	2,671	18.8
Associate Degree	2,105	14.9
Bachelor's Degree	7,021	49.5
Master's Degree	1,661	11.7
Occupation		
Student	3,483	24.6
Unemployed	2,388	16.9
Retired	1,088	7.7
Employee	7,212	50.9
Physical activity during the pandemic/lockdown		
No	6,641	46.9
Yes	7,530	53.1
Do you have health insurance?		
No	8,161	57.6
Yes	6,010	42.4
Do you smoke?		
No	10,817	76.3
Yes	3,354	23.7
Underlying medical condition		
Respiratory disease	1,478	12.1
Blood disease	434	2.8
Cancerous tumors	72	0.5
Diabetes	801	6.0
None	11,931	79.1
Underlying Psychological Disease		
No	12,716	89.7
Yes	1,455	10.3
Lockdown		
No Lockdown	6,052	42.7
<7 days	266	1.9
7 to 14 days	1,472	10.4
14 to 21 days	1,712	12.1
21 to 30 days	670	4.7
More than 30 days	3,999	28.2
Have you ever tested positive COVID-19?		
No	13,688	96.6
Yes	483	3.4

In this survey, the participants were also asked about their current occupational status. Out of the 14,171 individuals, more than half (50.9%) were employed, 24.6% were students, 16.9% unemployed, and 7.7% were retired.

In addition to sociodemographic characteristics, information about participants' health conditions was collected. About half of the respondents (53.1%) reported having physical activity during the pandemic/lockdown. 76.3% of participants were non-smokers, and 57.6% were not covered by any health insurance. Two thousand and seven hundred and eighty five individuals (21.4%) reported suffering from an underlying medical condition, 53.1% of whom had respiratory diseases, 28.7% suffered from diabetes, 15.6% from a blood disease, and 2.6% had cancerous tumors. In addition, 1,455 of all participants in this survey (10.3%) reported having an underlying psychological disease.

In pandemic-related questions, the participants were asked about the lockdown and Covid-19 infection. Six thousand and fifty two (42.7%) of participants did not experience any lockdown. 1.9% were in lockdown for <7 days, while 27.1% of respondents were in lockdown between 7 and 30 days, and 28.2% experienced lockdown for more than 30 days. Until that point, 483 respondents (3.4%) were infected with COVID-19 at least once.

#### Mental Health Condition of the Sample Population

Depression was the most common complication of the recent COVID-19 pandemic. As shown in Table 2, 80.1% of all participants in GCC countries felt depressed, while 1 in 4 suffered from severe depression. Only 20% experienced no symptoms of depression. The prevalence of depression was lowest among participants from Oman (65.9%). Severe symptoms of depression were also infrequent among Omani participants (13.7%). Feeling anxious was also very common among the participants (67.3%). 18.3% of participants in GCC countries suffered from severe anxiety, while around half of the population (49%) reported mild to moderate anxiety. The prevalence of a severe form of anxiety was highest in UAE with 25.5% and lowest among Omani respondents with 9.5%. Insomnia was less problematic than other mental health issues. In total, only 7.6% of participants in GCC countries reported severe insomnia symptoms, and 36.2% did not show any sign of insomnia. More than half of the respondents in Oman did not report any signs of insomnia, while only 2.9% reported severe insomnia symptoms. Severe sleeping problems were higher among participants in UAE (8.6%) compared to other GCC countries. 65.2% of all participants in GCC countries also showed signs of post-traumatic distress, 50.2% reported mild to moderate levels, and 5% had high levels of post-traumatic stress. With a prevalence of 55.2%, participants in Oman were the least affected by post-traumatic distress. The severe form of post-traumatic stress showed to be rare in all GCC countries.

**Table 2:** Frequency and prevalence of different severity levels of mental health issues in GCC countries.

**Mental health questionnaires**	**Total N (%)**	**UAE**	**Kuwait**	**KSA**	**Oman**	**Qatar**	**Bahrain**
PHQ-9 (Depression)	11,352 (80.1%)	778 (82.2%)	7,489 (82.3%)	731 (80.4%)	911 (65.9%)	624 (76.9%)	819 (80.1%)
No Depression	2,819 (19.9%)	168 (17.8%)	1,610 (17.7%)	178 (19.6%)	472 (34.1%)	187 (23.1%)	204 (19.9%)
Mild Depression	4,430 (31.3%)	262 (27.7%)	2,877 (31.6%)	261 (28.7%)	482 (34.9%)	243 (30%)	305 (29.8%)
Moderate Depression	3,373 (23.8%)	216 (22.8%)	2,279 (25%)	206 (22.7%)	240 (17.4%)	184 (22.7%)	248 (24.2%)
Severe Depression	3,549 (25%)	300 (31.7%)	2,333 (25.6%)	264 (29%)	189 (13.7%)	197 (24.3%)	266 (26%)
GAD-7 (Anxiety)	9,544 (67.3%)	673 (71.1%)	6,217 (68.3%)	647 (71.2%)	743 (53.7%)	535 (66%)	729 (71.3%)
No Anxiety	4,627 (32.7%)	273 (28.9%)	2,882 (31.7%)	262 (28.8%)	640 (46.3%)	276 (34%)	294 (28.7%)
Mild Anxiety	4,960 (35%)	304 (32.1%)	3,227 (35.5%)	311 (34.2%)	458 (33.1%)	286 (35.3%)	374 (36.6%)
Moderate Anxiety	1,991 (14%)	128 (13.5%)	1,307 (14.4%)	140 (15.4%)	153 (11.1%)	119 (14.7%)	144 (14.1%)
Severe Anxiety	2,593 (18.3%)	241 (25.5%)	1,683 (18.5%)	196 (21.6%)	132 (9.5%)	130 (16%)	211 (20.6%)
ISI-7 (Insomnia)	8,845 (63.9%)	631 (66.7%)	5,816 (63.9%)	585 (64.4%)	670 (48.4%)	498 (61.4%)	645 (63%)
No Insomnia	5,326 (36.2%)	315 (33.3%)	3,283 (36.1%)	324 (35.6%)	713 (51.6%)	313 (38.6%)	378 (37%)
Mild Insomnia	4,630 (32.9%)	326 (34.5%)	2,994 (32.9%)	281 (30.9%)	439 (31.7%)	262 (32.3%)	328 (32.1%)
Moderate Insomnia	3,221 (23.4%)	224 (23.7%)	2,128 (23.4%)	252 (27.7%)	191 (13.8%)	176 (21.7%)	250 (24.4%)
Severe Insomnia	994 (7.6%)	81 (8.6%)	694 (7.6%)	52 (5.7%)	40 (2.9%)	60 (7.4%)	67 (6.5%)
IES-R (Impact of Event scale)	9,046 (65.2%)	601 (63.5%)	5,932 (65.2%)	600 (66%)	764 (55.2%)	503 (62%)	646 (63.1%)
No Symptom	5,125 (34.7%)	345 (36.5%)	3,167 (34.8%)	309 (34%)	619 (44.8%)	308 (38%)	377 (36.9%)
Low level of Symptoms	3,992 (28.2%)	260 (27.5%)	2,560 (28.1%)	261 (28.7%)	394 (28.5%)	237 (29.2%)	280 (27.4%)
Moderate level of Symptoms	4,370 (32%)	293 (31%)	2,918 (32.1%)	291 (32%)	323 (23.4%)	235 (29%)	310 (30.3%)
High level of Symptoms	684 (5%)	48 (5.1%)	454 (5%)	48 (5.3%)	47 (3.4%)	31 (3.8%)	56 (5.5%)

*KSA, Kingdom of Saudi Arabia; UAE, United Arab Emirates*.

### Depression

The results of simple and multivariable logistic regressions in Table 3 demonstrate the association between predictor variables and depression. Generally, participants in Oman were significantly less likely to be depressed than other GCC countries, even when the potential confounding effects are controlled. According to the results, individuals in UAE are 1.39 (1.120–1.743), in Kuwait 1.67 (1.448–1.928), and in Bahrain 1.47 (1.194–1.812) times more likely to be depressed compared to individuals in Oman.

**Table 3:** Multivariable logistic regression assessing the association between different potential risk factors and mental health.

**VARIABLES**	**DEPRESSION**			**ANXIETY**			**INSOMNIA**			**POST-TRAUMATIC STRESS**		
	**N (%)**	**COR**	**AOR**	**N (%)**	**COR**	**AOR**	**N (%)**	**COR**	**AOR**	**N (%)**	**COR**	**AOR**
**Country**												
UAE	778 (82.2)	**2.399 (1.964–2.932)**	**1.398 (1.12–1.743)**	673 (71.1)	**2.123 (1.781–2.532)**	**1.397 (1.154–1.689)**	631 (66.7)	**2.132 (1.796–2.53)**	**1.455 (1.209–1.75)**	601 (63.5)	**1.411 (1.191–1.672)**	0.885 (0.737–1.063)
Kuwait	7489 (82.3)	**2.41 (2.13–2.727)**	**1.671 (1.448–1.928)**	6217 (68.3)	**1.858 (1.657–2.084)**	**1.411 (1.241–1.604)**	5816 (63.9)	**1.885 (1.682–2.112)**	**1.412 (1.244–1.602)**	5932 (65.2)	**1.518 (1.353–1.702)**	1.041 (0.917–1.183)
KSA	731 (80.4)	**2.128 (1.746–2.594)**	1.124 (0.904–1.397)	647 (71.2)	**2.127 (1.78–2.542)**	**1.335 (1.103–1.616)**	585 (64.4)	**1.921 (1.618–2.282)**	**1.22 (1.015–1.467)**	600 (66)	**1.573 (1.323–1.871)**	0.966 (0.802–1.163)
Qatar	624 (76.9)	**1.729 (1.419–2.107)**	**1.283 (1.036–1.589)**	535 (66)	**1.67 (1.395–1.998)**	**1.244 (1.028–1.505)**	498 (61.4)	**1.693 (1.419–2.02)**	**1.362 (1.13–1.64)**	503 (62)	**1.323 (1.108–1.579)**	0.926 (0.768–1.116)
Bahrain	819 (80.1)	**2.08 (1.721–2.514)**	**1.471 (1.194–1.812)**	729 (71.3)	**2.136 (1.799–2.536)**	**1.588 (1.321–1.908)**	645 (63)	**1.816 (1.54–2.142)**	**1.383 (1.16–1.649)**	646 (63.1)	**1.388 (1.177–1.638)**	0.971 (0.814–1.158)
Oman	911 (65.9)	Reference	Reference	743 (53.7)	Reference	Reference	670 (48.4)	Reference	Reference	764 (55.2)	Reference	Reference
**Gender**												
Female	8053 (84.4)	**2.199 (2.021–2.393)**	**1.858 (1.674–2.062)**	6919 (72.6)	**2.024 (1.881–2.179)**	**1.976 (1.808–2.159)**	6346 (66.5)	**1.7 (1.583–1.827)**	**1.549 (1.42–1.689)**	6617 (69.4)	**2.059 (1.915–2.213)**	**2.01 (1.844–2.191)**
Male	3299 (71.2)	Reference	Reference	2625 (56.6)	Reference	Reference	2499 (53.9)	Reference	Reference	2429 (52.4)	Reference	Reference
**Age**												
<18	485 (87.5)	**8.154 (5.175–12.846)**	**5.195 (2.974–9.074)**	399 (72)	**4.742 (3.065–7.336)**	**4.025 (2.416–6.708)**	383 (69.1)	**4.672 (3.004–7.264)**	**3.14 (1.886–5.226)**	379 (68.4)	**2.919 (1.915–4.449)**	**2.126 (1.294–3.492)**
18–24	3174 (89.8)	**10.199 (6.881–15.118)**	**5.223 (3.281–8.314)**	2612 (73.9)	**5.213 (3.487–7.792)**	**3.831 (2.432–6.035)**	2529 (71.5)	**5.243 (3.481–7.897)**	**3.286 (2.081–5.189)**	2406 (68.1)	**2.872 (1.949–4.233)**	**1.889 (1.213–2.941)**
25–34	3714 (83.1)	**5.684 (3.863–8.363)**	**3.759 (2.443–5.784)**	3153 (70.5)	**4.403 (2.951–6.57)**	**3.555 (2.3–5.495)**	2892 (64.7)	**3.818 (2.54–5.739)**	**2.972 (1.915–4.613)**	2879 (64.4)	**2.436 (1.655–3.584)**	**1.926 (1.259–2.947)**
35–44	2380 (76.2)	**3.716 (2.523–5.472)**	**2.729 (1.779–4.188)**	2071 (66.3)	**3.626 (2.426–5.42)**	**3.008 (1.949–4.644)**	1857 (59.5)	**3.059 (2.032–4.606)**	**2.522 (1.627–3.911)**	1957 (62.7)	**2.262 (1.534–3.335)**	**1.956 (1.28–2.99)**
45–54	1166 (68.3)	**2.495 (1.687–3.692)**	**1.984 (1.303–3.02)**	965 (56.5)	**2.393 (1.594–3.592)**	**2.032 (1.324–3.118)**	875 (51.2)	**2.191 (1.448–3.314)**	**1.867 (1.211–2.879)**	1031 (60.4)	**2.053 (1.385–3.042)**	**1.855 (1.22–2.82)**
55–64	383 (57.1)	**1.543 (1.026–2.32)**	1.358 (0.885–2.083)	306 (45.6)	**1.544 (1.012–2.358)**	1.373 (0.887–2.126)	274 (40.8)	1.44 (0.935–2.216)	1.291 (0.829–2.009)	348 (51.9)	1.452 (0.963–2.189)	1.337 (0.872–2.05)
> 65	50 (46.3)	Reference	Reference	38 (35.2)	Reference	Reference	35 (32.4)	Reference	Reference	46 (42.6)	Reference	Reference
**Marital status**												
Divorced	579 (82.1)	**1.636 (1.339–1.998)**	**1.317 (1.065–1.627)**	483 (68.5)	**1.252 (1.06–1.479)**	0.999 (0.838–1.191)	470 (66.7)	**1.53 (1.299–1.802)**	**1.315 (1.108–1.562)**	520 (73.8)	**1.925 (1.617–2.292)**	**1.544 (1.288–1.85)**
Single	5525 (86.9)	**2.358 (2.154–2.581)**	**1.166 (1.029–1.322)**	4556 (71.6)	**1.455 (1.352–1.565)**	0.934 (0.842–1.036)	4344 (68.3)	**1.649 (1.536–1.771)**	1.06 (0.96–1.17)	4281 (67.3)	**1.411 (1.314–1.515)**	**1.133 (1.025–1.251)**
Widowed	80 (69.6)	0.814 (0.545–1.215)	1.15 (0.747–1.77)	65 (56.5)	0.748 (0.516–1.085)	0.952 (0.64–1.416)	60 (52.2)	0.834 (0.577–1.207)	1.092 (0.738–1.617)	79 (68.7)	**1.503 (1.01–2.236)**	1.504 (0.992–2.28)
Separated	97 (83.6)	**1.817 (1.108–2.98)**	1.341 (0.804–2.238)	76 (65.5)	1.094 (0.743–1.609)	0.799 (0.535–1.193)	75 (64.7)	1.399 (0.953–2.053)	1.118 (0.752–1.663)	85 (73.3)	**1.878 (1.241–2.841)**	1.457 (0.953–2.227)
Married	5071 (73.7)	Reference	Reference	4364 (63.5)	Reference	Reference	3896 (56.7)	Reference	Reference	4081 (59.4)	Reference	Reference
**Health insurance**												
No	6602 (80.9)	**1.123 (1.034–1.221)**	0.964 (0.878–1.059)	5556 (68.1)	**1.081 (1.007–1.161)**	0.992 (0.918–1.072)	5146 (63.1)	1.066 (0.996–1.142)	0.979 (0.908–1.055)	5227 (64)	1.022 (0.954–1.095)	0.949 (0.88–1.024)
Yes	4750 (79)	Reference	Reference	3988 (66.4)	Reference	Reference	3699 (61.5)	Reference	Reference	3819 (63.5)	Reference	Reference
**Level of education**												
< HSD	552 (77.4)	1.098 (0.892–1.353)	**0.672 (0.522–0.865)**	472 (66.2)	1.131 (0.94–1.36)	0.875 (0.701–1.093)	447 (62.7)	**1.27 (1.061–1.521)**	0.918 (0.74–1.139)	454 (63.7)	1.17 (0.976–1.403)	0.974 (0.785–1.208)
HSD	2183 (81.7)	**1.433 (1.235–1.663)**	0.909 (0.768–1.076)	1828 (68.4)	**1.252 (1.101–1.424)**	0.961 (0.831–1.11)	1727 (64.7)	**1.383 (1.22–1.567)**	0.983 (0.855–1.131)	1683 (63)	**1.137 (1.003–1.29)**	0.98 (0.852–1.127)
AD	1599 (76)	1.012 (0.871–1.176)	**0.834 (0.709–0.982)**	1396 (66.3)	1.137 (0.994–1.301)	1.017 (0.881–1.175)	1275 (60.6)	**1.161 (1.019–1.323)**	1.02 (0.888–1.172)	1311 (62.3)	1.102 (0.966–1.258)	1.033 (0.899–1.188)
BSA	5760 (82)	**1.463 (1.288–1.663)**	1.064 (0.926–1.223)	4795 (68.3)	**1.244 (1.112–1.391)**	1.011 (0.896–1.14)	4450 (63.4)	**1.308 (1.174–1.458)**	1.054 (0.938–1.183)	4602 (65.5)	**1.27 (1.138–1.418)**	**1.137 (1.011–1.278)**
MSA	1258 (75.7)	Reference	Reference	1053 (63.4)	Reference	Reference	946 (57)	Reference	Reference	996 (60)	Reference	Reference
**Occupation**												
Student	3129 (89.8)	**2.542 (2.248–2.875)**	**1.351 (1.1–1.66)**	2574 (73.9)	**1.537 (1.405–1.681)**	1.163 (0.998–1.355)	2514 (72.2)	**1.828 (1.674–1.995)**	**1.422 (1.227–1.647)**	2405 (69)	**1.42 (1.303–1.548)**	**1.212 (1.048–1.402)**
Unemployed	1981 (83)	**1.4 (1.241–1.579)**	1.133 (0.989–1.297)	1757 (73.6)	**1.511 (1.363–1.675)**	**1.227 (1.095–1.376)**	1617 (67.7)	**1.478 (1.34–1.629)**	**1.305 (1.171–1.454)**	1635 (68.5)	**1.382 (1.252–1.525)**	**1.185 (1.063–1.321)**
Retired	641 (58.9)	**0.412 (0.361–0.471)**	**0.69 (0.576–0.826)**	538 (49.4)	**0.531 (0.467–0.604)**	0.839 (0.708–0.995)	483 (44.4)	**0.562 (0.495–0.64)**	0.861 (0.728–1.018)	599 (55.1)	**0.78 (0.686–0.887)**	0.909 (0.767–1.078)
Employee	5601 (77.7)	Reference	Reference	4675 (64.8)	Reference	Reference	4231 (58.7)	Reference	Reference	4407 (61.1)	Reference	Reference
**Exercise**												
No	5579 (84)	**1.599 (1.469–1.74)**	**1.628 (1.487–1.781)**	4736 (71.3)	**1.407 (1.311–1.511)**	**1.373 (1.274–1.479)**	4433 (66.8)	**1.419 (1.325–1.52)**	**1.41 (1.312–1.515)**	4374 (65.9)	**1.18 (1.102–1.264)**	**1.156 (1.076–1.242)**
Yes	5773 (76.7)	Reference	Reference	4808 (63.9)	Reference	Reference	4412 (58.6)	Reference	Reference	4672 (62)	Reference	Reference
**Smoke**
No	8600 (79.5)	**0.849 (0.768–0.938)**	**0.629 (0.56–0.707)**	7181 (66.4)	**0.828 (0.761–0.901)**	**0.623 (0.564–0.687)**	6607 (61.1)	**0.783 (0.721–0.849)**	**0.635 (0.578–0.698)**	6860 (63.4)	0.926 (0.854–1.005)	**0.691 (0.629–0.759)**
Yes	2752 (82.1)	Reference	Reference	2363 (70.5)	Reference	Reference	2238 (66.7)	Reference	Reference	2186 (65.2)	Reference	Reference
**Psychological disorder**												
No	1365 (93.8)	Reference	Reference	1235 (84.9)	Reference	Reference	1156 (79.5)	Reference	Reference	1231 (84.6)	Reference	Reference
Yes	9987 (78.5)	**4.144 (3.334–5.151)**	**3.208 (2.567–4.009)**	8309 (65.3)	**2.977 (2.568–3.452)**	**2.451 (2.106–2.853)**	7689 (60.5)	**2.528 (2.215–2.884)**	**2.098 (1.831–2.404)**	7815 (61.5)	**3.446 (2.976–3.991)**	**2.933 (2.525–3.406)**
**Underlying disease**												
No	2177 (81.7)	Reference	Reference	1901 (71.4)	Reference	Reference	1764 (66.2)	Reference	Reference	1837 (69)	Reference	Reference
Yes	9175 (79.7)	**1.139 (1.022–1.269)**	**1.317 (1.169–1.485)**	7643 (66.4)	**1.262 (1.15–1.384)**	**1.385 (1.254–1.531)**	7081 (61.5)	**1.227 (1.123–1.34)**	**1.34 (1.218–1.474)**	7209 (62.6)	**1.326 (1.212–1.452)**	**1.329 (1.207–1.462)**
**Lockdown**												
No and less than 7 days lockdown	4788 (75.8)	**0.523 (0.471–0.582)**	**0.689 (0.612–0.776)**	4087 (64.7)	**0.699 (0.642–0.763)**	**0.848 (0.77–0.934)**	3698 (58.5)	**0.682 (0.628–0.741)**	**0.818 (0.746–0.897)**	3836 (60.7)	**0.723 (0.665–0.786)**	**0.835 (0.761–0.916)**
7–30 days lockdown	3138 (81.4)	**0.733 (0.65–0.827)**	**0.754 (0.663–0.857)**	2563 (66.5)	**0.758 (0.688–0.835)**	**0.798 (0.721–0.883)**	2451 (63.6)	**0.844 (0.769–0.927)**	**0.888 (0.805–0.979)**	2486 (64.5)	**0.851 (0.775–0.934)**	**0.88 (0.798–0.971)**
> 30 days lockdown	3426 (85.7)	Reference	Reference	2894 (72.4)	Reference	Reference	2696 (67.4)	Reference	Reference	2724 (68.1)	Reference	Reference
**COVID-19 Test**												
Negative	10955 (80)	0.868 (0.685–1.1)	**0.736 (0.573–0.945)**	9210 (67.3)	0.918 (0.754–1.117)	**0.803 (0.654–0.985)**	8533 (62.3)	0.907 (0.75–1.097)	**0.819 (0.672–0.998)**	8738 (63.8)	1.003 (0.83–1.211)	0.885 (0.728–1.076)
Positive	397 (82.2)	Reference	Reference	334 (69.2)	Reference	Reference	312 (64.6)	Reference	Reference	308 (63.8)	Reference	Reference

*AOR, adjusted odds ratio; COR, crude odds ratio; CI, Confidence Interval; HSD, high school diploma; AD, associate degree; BSA, Bachelor's Degree; MSA, Master's Degree. Bold numbers: statistically significant at p < 0.05*.

The prevalence of depressive symptoms among females was 84.4 and 71.2% among males. Multivariable logistic regression showed that females are 86% more likely to exhibit depressive symptoms than males (AOR = 1.858; 1.674–2.062). Calculated adjusted odds ratios also showed that age is an important risk factor for depression during the pandemic. The odds of having depressive symptoms among participants younger than 25 years old were more than 5 times higher than individuals older than 65 (<18: AOR = 5.195; 2.974–9.074, 18–24: AOR = 5.223; 3.281–8.314). Divorced (AOR = 1.317; 1.065–1.627) and single (AOR = 1.166; 1.029–1.322) individuals were also more affected by depression than married people.

In addition, students were 35% more likely to show depressive symptoms (AOR = 1.351; 1.100–1.660), while retired individuals were 31% less likely to be depressed (AOR = 0.690; 0.576–0.826) compared to employed individuals. Participants who did not have physical activity during the pandemic were 62.8% more likely to show signs of depression (AOR = 1.628; 1.487–1.781). Non-smokers were also 47% less likely to have symptoms of depression (AOR = 0.629; 0.560–0.707) compared to smokers.

Furthermore, the odds of being depressed was 31.7% higher among individuals with an underlying medical condition (AOR = 1.317; 1.169–1.485) and 3.2 times higher among individuals with underlying psychological disorders (AOR = 3.208; 2.567–4.009). Regarding the duration of lockdown, individuals who experienced more than 30 days of lockdown were significantly more at risk of depression. Participants who were in no lockdown or experienced the lockdown for <7 days were 31% less likely to show depressive symptoms (AOR = 0.689; 0.612–0.776) than those who were in lockdown for more than 30 days. Finally, participants who were never infected with COVID-19 were 26.4% less likely to be depressed than those who contracted COVID-19 at least once.

### Anxiety

The association between predictor variables and anxiety was also investigated using multivariate logistic regression (Table 3). Similar to depression, Oman was significantly in a better situation regarding anxiety. Compared to Oman, participants in Bahrain were 58%, in Kuwait 41%, and in UAE about 40% more likely to be anxious during the pandemic. The prevalence of anxiety symptoms was 56.6% among males and 72.6% among females compared to males. Females had significantly higher adjusted odds of being anxious during the pandemic (AOR = 1.976; 1.808–2.159). The likelihood of being anxious was also significantly higher among younger people compared to individuals older than 65. For example, participants younger than 18 were 4 times more likely to show anxiety symptoms (AOR = 4.025; 2.416–6.708). Individuals between 55 and 64 did not differ significantly from those older than 65 (AOR = 1.373; 0.887–2.126).

According to the adjusted odds ratios, marital status, having health insurance, and education level appeared not to affect the likelihood of developing anxiety symptoms during the pandemic. Unemployment, however, does play an important role in the development of anxiety symptoms. Unemployed individuals showed higher adjusted odds of being anxious during the pandemic than employed individuals (AOR = 1.227; 1.095–1.376). Like depression, physical activity also significantly lowered the likelihood of experiencing anxiety symptoms, while individuals who did not have any physical activity during the pandemic were 37% more likely to be anxious (AOR = 1.373; 1.274–1.479). Non-smokers were also 37% less likely to have anxiety symptoms (AOR = 0.623; 0.564–0.687) than smokers. Participants with underlying psychological disorders were 2.4 times (AOR = 2.451; 2.106–2.853), and individuals with underlying medical conditions were 38% more likely to experience any symptom of anxiety (AOR = 1.385; 1.254–1.531).

Similar to depression, adjusted odds of being anxious was significantly lower among individuals who were under lockdown between 7 and 30 days (AOR = 0.798; 0.721–0.883) and <7 days (AOR = 0.848; 0.770–0.934) compared to those who experienced the lockdowns for more than 30 days. Individuals without COVID-19 infection were also 20% less likely to experience anxiety during the pandemic (AOR = 0.803; 0.654–0.985).

### Insomnia

The prevalence of Insomnia among Omani participants was 48.4%, while this was above 60% among other GCC countries. Participants from UAE, with 66.7%, suffered more from insomnia. According to the adjusted odds ratios calculated in multivariable logistic regression in Table 3, participants in UAE (AOR = 1.455; 1.209–1.750) and Kuwait (AOR = 1.412; 1.244–1.602) were 40% more likely to show insomnia symptoms than participants in Oman. Females were also shown to be more susceptible to insomnia during the pandemic since they were 55% more likely to report sleeping problems compared to males (AOR = 1.549; 1.420–1.689). Similar to depression and anxiety, this study shows a powerful reverse association between age and insomnia. All age groups younger than 35 years old were about 3 times more likely to show insomnia symptoms than age group >65. The most susceptible age group was shown to be 18 to 24 years old with an adjusted odds ratio of 3.286 (1.209–1.750). Regarding marital status, divorced individuals were 31.5% more likely to have sleeping problems than married individuals. Similar to anxiety, having health insurance and the level of education did not affect the likelihood of showing insomnia symptoms compared to employed individuals, students (AOR = 1.422; 1.227–1.647) were 42%, and unemployed individuals (AOR = 1.305; 1.171–1.454) were 30% more likely to experience sleeping problems. Similar to depression and anxiety, physical activity decreased the likelihood of reporting insomnia symptoms by 29% (AOR = 1.410; 1.312–1.515). Non-smokers were also 36.5% less likely to report any sign of insomnia than smokers (AOR = 0.635; 0.578–0.698). Furthermore, participants with underlying psychological disorders were 2.1 times (AOR = 2.098; 1.831–2.404) and individuals with underlying medical conditions 34% more likely to experience any symptom of insomnia (AOR = 1.340; 1.218–1.474). Regarding the duration of lockdown, participants who were in no lockdown or experienced the lockdown for <7 days were 18.2% less likely to show insomnia symptoms (AOR = 0.818; 0.746–0.897) compared to those who were in lockdown for more than 30 days. Finally, participants who were never infected with COVID-19 were 18.1% less likely to experience sleep problems than those who contracted COVID-19 at least once (AOR = 0.819; 0.672–0.998).

### Post-traumatic Stress

Finally, the association between predictor variables and post-traumatic stress was investigated using multivariable logistic regression (Table 3). Although crude odds ratios indicated a strong association between the country and post-traumatic stress, this association disappears when the potential confounding effects are considered (adjusted odds ratios). Hence, participants in all GCC countries seem to have a similar likelihood to experience post-traumatic stress during the pandemic when all other predictor variables are controlled.

The prevalence of post-traumatic stress symptoms was 52.4% among males and 69.4% among females. Females, compared to men, had significantly higher adjusted odds of being stressed during the pandemic (AOR = 2.010; 1.844–2.191). The likelihood of having post-traumatic stress was also significantly higher among younger people compared to individuals older than 65. For example, participants younger than 18 were 2.1 times more likely to show stress symptoms (AOR = 2.126; 1.294–3.492). Individuals between 55 and 64 did not differ significantly from those older than 65 (AOR = 1.337; 0.872–2.050).

According to the adjusted odds ratios, having health insurance and COVID-19 infection appeared not to affect the likelihood of developing post-traumatic stress symptoms during the pandemic. Occupation, however, does play an important role in the development of post-traumatic stress. Unemployed individuals (AOR = 1.185; 1.063–1.321) and students (AOR = 1.212; 1.048–1.402) showed higher adjusted odds of being stressed during the pandemic than employed individuals. In regard to marital status, divorced individuals were 54.4% (AOR = 1.544; 1.288–1.850), Singles 13.3% (AOR = 1.133; 1.025–1.251) more likely to suffer from post-traumatic stress compared to married individuals.

In addition to depression, anxiety, and insomnia, physical activity also lowers the likelihood of experiencing stress symptoms. Thus, individuals who did not have any physical activity during the pandemic were 15.6% more likely to suffer from post-traumatic stress (AOR = 1.156; 1.076–1.242). Non-smokers were also 31% less likely to have symptoms of post-traumatic stress (AOR = 0.691; 0.629–0.759) than smokers. Participants with underlying psychological disorders were 2.9 times (AOR = 2.933; 2.525–3.406), and individuals with underlying medical conditions were 33% more likely to experience any symptom of post-traumatic stress (AOR = 1.329; 1.207–1.462).

The results also showed that individuals who were under lockdown between 7 and 30 days (AOR = 0.880; 0.798–0.971) and <7 days (AOR = 0.835; 0.761–0.916) were less likely to report any sign of post-traumatic stress compared to those who experienced the lockdowns for more than 30 days.

## Discussion

During the COVID-19 pandemic, the population in many countries was negatively affected. Many sociodemographic, health, and pandemic-related factors, such as unemployment, uncertainty, distress, increasing deaths, and lockdown measures, have been potential burdens on mental health. In this study, 14,171 participants from GCC countries were asked to self-assess their mental health in four areas, including depression, anxiety, insomnia, and post-traumatic stress level. The COVID-19 pandemic had a significant impact on the mental health of GCC countries. However, this effect has not been equal across all 6 countries. In general, Oman has been shown to have fewer self-reported depression, anxiety, and insomnia cases than other GCC countries, while the prevalence of post-traumatic stress among all GCC countries was similar.

The current study indicated that females are disproportionately susceptible to depression, anxiety, and post-traumatic distress. This finding is similar to the reports of other population-based studies assessing the burden of mental health conditions during the COVID-19 pandemic (33, 34). In addition, a comprehensive meta-analysis conducted by Zeng et al. (35) demonstrated that the prevalence of insomnia among females was significantly higher than males in the included studies, which is also similar to the results of this study. Besides, restrictive measures, such as school closures, could be the reason behind the increase of the burden on women at home, leading to tiredness and decreased work performance (36, 37).

It is already known that different age groups react differently to environmental stress. Numerous studies have indicated age to be one of the main risk factors significantly impacting mental health susceptibility. A meta-analysis conducted by Bonanad et al. (38) highlighted the importance of older age on mortality. There were also concerns about the disruption of daily lives and challenges that they have using technologies. Hence, it was expected that older people, especially > 60, are at higher risk of experiencing mental health issues. In this study, however, the results showed the opposite. Individuals older than 55 were significantly more resilient to depression, anxiety, insomnia, and post-traumatic stress. These findings are similar to the result of a study conducted by Vahia et al. (39). The reason behind this resilience needs further investigation. However, a study conducted by Laird et al. (40) indicated that this resilience could reflect internal factors, such as biological stress response, cognitive capacity, personality traits, or external factors, such as financial stability and social status.

As expected, individuals younger than 24 years old in GCC countries were strongly prone to depression. Similarly, anxiety and insomnia were much more common among younger individuals. However, the likelihood of post-traumatic distress was more or less similar among different age groups younger than 55 years old compared to individuals older than 65.

Menon et al. (41) had demonstrated that loneliness is one of the main consequences of COVID-19 pandemics and subsequent studies confirmed that lonely people are significantly more vulnerable to anxiety, depression, and post-traumatic stress (42). In agreement with the aforementioned researches, the current study found that single and especially divorced individuals were significantly more prone to depression and post-traumatic stress than married individuals. Additionally, this study demonstrated that divorced individuals were also more likely to suffer from insomnia. However, in contrast to previous studies, there was no association between marital status and anxiety, even among divorced and single people.

Another finding of this study was the positive impact of physical activity on overall mental health. As indicated in the results section, physical activity during the pandemic significantly decreased individuals' likelihood of depression, anxiety, and post-traumatic stress. In addition, it notably lowered the prevalence of depressive symptoms. This finding is also supported by a study conducted in the UK, which demonstrated that physical activity during the COVID-19 pandemic counteracts the negative effects of coronavirus, namely fear and stress, especially among young people (43).

Previous studies have indicated that depression plays a crucial role in cigarette smoking habits (44). In addition, Loren et al. (45) demonstrated that smoking is strongly associated with poor mental health, including psychological distress. Furthermore, research has shown that smoking increases anxiety (46). The finding in this study is similar to previous studies, indicating that smoking and mental health issues are strongly associated when gender as a confounding effect is controlled and speculating that nicotine increases the risk of anxiety and post-traumatic stress symptoms. Interestingly, smokers who participated in this study were significantly more likely to suffer from sleep disturbances than non-smokers. This can be explained by the study conducted by Nunez et al. (47), providing evidence that smoking is associated with increased insomnia severity and shorter sleep duration, particularly nightly smoking. The association between smoking and depression could also be demonstrated in this study. This association was also observed in previous studies (48).

One of the most crucial findings of this study was the strong vulnerability of individuals with underlying psychological disorders against mental health issues. It was shown that they are significantly more likely to suffer from anxiety, insomnia, post-traumatic distress, and particularly depression. In agreement with this study, Neelam et al. (49) demonstrated in a meta-analysis that people with pre-existing mental illnesses experience high levels of anxiety, depression, post-traumatic stress, and sleep problems during the pandemic. However, it needs further investigation to see whether this is due to the pandemic or simply reflects higher levels of symptoms in a clinical population.

Another objective of this study was to investigate the impact of lockdown and lockdown duration. Previous studies (50, 51) have already demonstrated that lockdowns had an immediate negative impact on the mental health of general populations worldwide. The results of this study are consistent with these findings, indicating that symptoms of depression, anxiety, insomnia, and post-traumatic stress were significantly more prevalent among individuals who were in lockdown for more than 30 days. Concerning positive COVID-19 diagnosis, previous research has already shown that COVID-19 patients experience significant psychological distress (52). This finding is also consistent with the results of this study among participants in GCC countries. This study found that confirmed COVID-19 diagnosis increases the likelihood to report depression, anxiety, and sleeping problems, while no significant increase in post-traumatic distress was observed among positively diagnosed participants. Indeed, this contradicts a study conducted by Tarsitani et al. (53) and Bridgland et al. (54), suggesting an association between COVID-19 diagnosis and post-traumatic stress.

The outcome of this study suggests that several groups are particularly more vulnerable to depression, anxiety, insomnia, and post-traumatic stress. These findings should enable the policy-makers to prioritize their attention toward those who are most affected by the pandemic and lockdowns. Furthermore, it helps them to make informed adjustments to restrictive measures. Younger individuals, especially children, should be given special attention since they are highly prone to depression, anxiety, insomnia, and post-traumatic stress. In addition, facilitating plans for daily physical activity during lockdown is one of the most critical measures that can counteract the negative impact of a pandemic. People with pre-existing psychological disorders also need special care. Hence, the health and education authorities should provide more support to institutions, such as schools, sports organizations, and mental health organizations, to minimize the negative impacts of lockdowns.

Furthermore, authorities should consider allocating more resources to support those groups of people who are particularly at risk of being lonely, like singles and divorced individuals. For example, according to a cross-sectional study conducted by Agyapong et al. (55), supportive text messages effectively reduce depressive symptoms. They are independent of geographic location, are accessible to the end-users, do not require expensive data plans, and can simultaneously reach thousands of people.

The strength of this study is its large sample, including 14,171 participants of the GCC countries. The four questionnaires about depression (PHQ-9), anxiety (GAD-7), insomnia (ISI-7), and post-traumatic stress (IES-R-22) are all validated in different languages and have been utilized in the field of mental health for a long time. As the pandemic progresses, additional efforts are needed to track the effects of the pandemic and lockdowns on the mental health of the public. There will probably be new waves of this disease. Accordingly, additional restrictive measures will be introduced. Longitudinal studies are needed to track and observe the gradual change of mental health with changing restrictive measures.

Naturally, there are also some limitations to this study. First, this was mainly a cross-sectional study which cannot imply causation. Secondly, the survey began late during the first restrictive measures/lockdowns, so the participant's pre-pandemic and pre-lockdown mental health conditions could not be known. Furthermore, the survey was sent to the respondents online, which could disproportionately exclude the individuals who either did not have access to or were uncomfortable using the internet. In addition, this study was conducted by a self-report questionnaire, which is usually prone to response bias. Finally, the generalizability of this study is also limited due to the convenience/snowball sampling approach. Thus, future comprehensive longitudinal studies are needed to support or dispute the findings of this study.

In conclusion, the present study found that many sociodemographic characteristics can be associated with a higher risk of mental health issues in the general population. Women, youth, and people with pre-existing psychological and medical disorders were particularly susceptible to a higher risk of mental health problems during the pandemic and lockdown. In addition, being single and divorced, which is usually associated with being lonely, could be considered predictors of depression and post-traumatic stress. According to the results, regular physical activity could be recommended to relatively compensate for the negative impact of COVID-19 on the mental health and well-being of the public. These findings highlight that several groups are particularly vulnerable to depression, anxiety, insomnia, and post-traumatic stress, which policy-makers should seriously consider when introducing or adjusting restrictive measures during the pandemic.

## Data Availability Statement

The raw data supporting the conclusions of this article will be made available by the authors, without undue reservation.

## Ethics Statement

The studies involving human participants were reviewed and approved by Health Sciences Centre (HSC) Ethical Committee of Kuwait University. Written informed consent to participate in this study was provided by the participants' legal guardian/next of kin.

## Author Contributions

NaA-M designed and conceptualized the study, contributed to the data analysis and formulation of the manuscript, and also drafted the manuscript. NoA-M contributed to the literature review, data collection, and manuscript editing. Both authors contributed to the article and approved the submitted version.

## Conflict of Interest

The authors declare that the research was conducted in the absence of any commercial or financial relationships that could be construed as a potential conflict of interest.

## Publisher's Note

All claims expressed in this article are solely those of the authors and do not necessarily represent those of their affiliated organizations, or those of the publisher, the editors and the reviewers. Any product that may be evaluated in this article, or claim that may be made by its manufacturer, is not guaranteed or endorsed by the publisher.
