# Supplementary material for: Impact of COVID-19 Pandemic and Lockdown Measures on the Mental Health of the General Population in the Gulf Cooperation Council States: A Cross-Sectional Study
Source: Front Psychiatry. 2021 Dec 20;12:801002. doi: 10.3389/fpsyt.2021.801002 (PMC8721202; doi:10.3389/fpsyt.2021.801002)
Supplement: Supplementary file 1 [file Data_Sheet_1.PDF]

Generalized Anxiety Disorder 7-item (GAD-7) scale

Over the last 2 weeks, how often have you been bothered by the following problems?	Not at all sure	Several days	Over half the days	Nearly every day
1. Feeling nervous, anxious, or on edge	0	1	2	3
2. Not being able to stop or control worrying	0	1	2	3
3. Worrying too much about different things	0	1	2	3
4. Trouble relaxing	0	1	2	3
5. Being so restless that it's hard to sit still	0	1	2	3
6. Becoming easily annoyed or irritable	0	1	2	3
7. Feeling afraid as if something awful might happen	0	1	2	3
Add the score for each column	+	+	+	
Total Score *(add your column scores)* =				

If you checked off any problems, how difficult have these made it for you to do your work, take care of things at home, or get along with other people?

Not difficult at all __________
Somewhat difficult _________
Very difficult _____________
Extremely difficult _________

Source: Spitzer RL, Kroenke K, Williams JBW, Lowe B. A brief measure for assessing generalized anxiety disorder. *Arch Inern Med.* 2006;166:1092-1097.

Interpreting the Score:

Total Score	Interpretation
≥10	Possible diagnosis of GAD; confirm by further evaluation
5	Mild Anxiety
10	Moderate anxiety
15	Severe anxiety
