# Supplementary material for: Impact of COVID-19 Pandemic and Lockdown Measures on the Mental Health of the General Population in the Gulf Cooperation Council States: A Cross-Sectional Study
Source: Front Psychiatry. 2021 Dec 20;12:801002. doi: 10.3389/fpsyt.2021.801002 (PMC8721202; doi:10.3389/fpsyt.2021.801002)
Supplement: Supplementary file 2 [file Data_Sheet_2.PDF]

impact of event scale - revised

your name: *today's date:*

on _______ you experienced ________________<br>(date) (life event)<br>below is a list of difficulties people sometimes have after stressful life events. please read each item and then indicate how distressing each difficulty has been for you *during the past 7 days or other agreed time:*		*how distressing?*<br>*not at all* 0 · *a little bit* 1 · *moder -ately* 2 · *quite a bit* 3 · *extre -mely* 4
a.	any reminder brought back feelings about it	
b.	I had trouble staying asleep	
c.	other things kept making me think about it	
d.	I felt irritable and angry	
e.	I avoided letting myself get upset when I thought about it or was reminded of it	
f.	I thought about it when I didn't mean to	
g.	I felt as if it hadn't happened or it wasn't real	
h.	I stayed away from reminders about it	
i.	pictures about it popped into my mind	
j.	I was jumpy and easily startled	
k.	I tried not to think about it	
l.	I was aware that I still had a lot of feel-ings about it, but I didn't deal with them	
m.	my feelings about it were kind of numb	
n.	I found myself acting or feeling like I was back at that time	
o.	I had trouble falling asleep	
p.	I had waves of strong feelings about it	
q.	I tried to remove it from my memory	
r.	I had trouble concentrating	
s.	reminders of it caused me to have phys-ical reactions, such as sweating, trouble breathing, nausea. or a pounding heart	
t.	I had dreams about it	
u.	I felt watchful and on-guard	
v.	I tried not to talk about it	

total score

avoidance subscale (total of e, g, h, k, l, m, q, v divided by 8) =

intrusion subscale (total of a, b, c, f, i, n, p, t divided by 8) =

hyperarousal subscale (total of d, j, o, r, s, u divided by 6) =

Weiss D.S. & Marmar C.R. *The impact of event scale-revised.* in Wilson J.P. & Kean T.M. (eds.) *Assessing psychcological trauma and PTSD: a practitioner's handbook (ch 15).* N.Y: Guildford, 1995.
